# ^1^H, ^13^C, ^15^N backbone chemical shift assignments of the extended ARID domain in human AT-rich interactive domain protein 5a (*Arid5a*)

**DOI:** 10.1007/s12104-023-10130-w

**Published:** 2023-05-02

**Authors:** Julian von Ehr, Sophie Marianne Korn, Lena Weiß, Andreas Schlundt

**Affiliations:** 1 Institute for Molecular Biosciences and Biomolecular Resonance Center (BMRZ) of Goethe University Frankfurt, Max-von-Laue-Str. 7-9, 60438 Frankfurt am Main, Germany; 2 IMPRS on Cellular Biophysics, Max-von-Laue-Str. 7-9, 60438 Frankfurt am Main, Germany

**Keywords:** AT-rich interactive domain containing proteins, ARID domain, Nucleic acid-binding, Helix-turn-helix motif, Transcription regulation, Inflammatory diseases

## Abstract

The family of AT-rich interactive domain (ARID) containing proteins -*Arids*- contains 15 members that have almost exclusively been described as DNA-binding proteins. Interestingly, a decade ago the family member *Arid5a* was found to bind and stabilize mRNAs of immune system key players and thereby account for driving inflammatory and autoimmune diseases. How exactly binding to DNA and RNA is coordinated by the *Arid5a* ARID domain remains unknown, mainly due to the lack of atom-resolved information on nucleic acid-binding. This in particular applies to the protein’s ARID domain, despite the comfortable size of its core unit for NMR-based investigations. Furthermore, the core domain of ARID domains is found to be extended by functionally relevant, often flexible stretches, but whether such elongations are present and crucial for the versatile *Arid5a* functions is unknown. We here provide a near-complete NMR backbone resonance assignment of the *Arid5a* ARID domain with N- and C-terminal extensions, which serves as a basis for further studies of its nucleic acid-binding preferences and targeted inhibition by means of NMR. Our data thus significantly contribute to unravelling mechanisms of *Arid5a*-mediated gene regulation and diseases.

## Biological context

Within any organism, a plethora of nucleic acid-binding proteins (NBPs) exert a wide variety of functions on different levels of gene regulation. These proteins sub-classify into DNA-binding proteins (DBP) (Brennan and Matthews 1989a), RNA-binding proteins (RBP) (Re et al. 2014), and those that can bind either type of nucleic acid, so-called DNA/RNA-binding proteins (DRBPs) (Hudson and Ortlund 2014). To exhibit their respective functions, NBPs exploit specialized domains, likewise classified as DNA- or RNA-binding domains (DBD, RBD). One of the most common and central functional units for nucleic acid-recognition within them is the helix-turn-helix (HTH) motif (Aravind et al. 2005). The HTH consists of two α-helices connected by a short loop and is primarily found in DBDs, but is in principle competent in recognition of dsRNA (Schuetz et al. 2014). Yet, to our best knowledge, the precise determinants for the discrimination of DNA and RNA have so-far not been investigated in structural detail.

The human AT-rich interactive domain (ARID) containing proteins -termed *Arids-* comprise a family of 15 members, which are grouped into seven sub-families and share the characteristic appearance of an HTH-motif as part of the name-giving ARID domain (Gregory et al. 1996; Herrscher et al. 1995; Yuan et al. 1998). ARID domains are approximately 100 amino acids in size, and their core fold comprises six α-helices (H1 to H6). Within ARIDs, the helices H1 and H2 are consistently connected by an extended loop (L1). A second loop (L2) is found between H3/H4 and H5, which all together were found to be the responsible HTH motif for interacting with dsDNA (Kim et al. 2004; Cai et al. 2007). Importantly, the HTH in *Arids* shows a non-canonical, atypically long L2 (Iwahara and Clubb 1999) as compared to canonical HTHs with a 3-amino acid loop (Brennan and Matthews 1989b).

Variants of the core ARID domain exist with extensions by additional helices, either located N- (H0) and/or C-terminally (H7) (Kortschak et al. 2000). These extensions can be involved in further contacts with DNA and thereby influence the binding of protein to DNA in terms of affinity and specificity (Liu et al. 2010; Iwahara and Clubb 1999). Moreover, intrinsically disordered regions (IDRs) flanking the core domain will potentially contribute to DNA-recognition in a nonspecific manner (Korn and Schlundt 2022). How exactly the various types of ARIDs exhibit their different specificities to DNA remains unknown. This is majorly due to a lack of high-resolution structural information of ARIDs in complex with target DNAs, in particular including the flexible overhangs. Consequently, the precise boundaries of core ARID domains and their possible extensions appear as a first starting point for investigating their DNA-binding capacities and specificities. To this end, NMR spectroscopy provides the ideal output in mapping and quantifying interactions at residue-to-atom-level. Beyond the valuable information of complex structures, NMR chemical shifts aid in examining the contributions of ARID domain elements to DNA-binding even in the absence of high-resolution structures and will thus provide precise information at the structural level, e.g. used in systematic comparison between ARID domains and their target DNAs.

The *Arid*-family member 5a has recently come into focus as a DRBP (Masuda et al. 2013), proven by its RNA-binding capability. This finding makes it unique among *Arids*, considering all other proteins -to date- are described to exclusively bind DNA. It makes *Arid5a* an intriguing target to study in order to decipher its exclusive RBP character among *Arids*, and raises the question of a more general dual nucleic acid-binding competence among *Arids.* The *Arid5a* RNA-binding capabilities have been linked to immune-regulatory roles on the transcript level. Here, the protein was suggested to bind to stem-loop decay elements and protect the mRNA from degradation. In competing with previously described mRNA-repressive RBPs (Hanieh et al. 2018; Masuda et al. 2016; Parajuli et al. 2021) *Arid5a* was thus categorized as a pro-inflammatory immune-regulator and important future drug target (Nyati et al. 2019).

Interestingly, the structure of the closely related DNA-binding *Arid5b* ARID domain (Protein Data Bank (PDB) ID: 2OEH, Cai et al. 2007) reveals a canonical core domain, but possible extensions have been ignored despite a high degree of *Arid5b* sequence conservation between species, as well as a reasonable conservation between 5a and b. Whether and how additional segments contribute to DNA- and RNA-binding including a possible discriminative potency via the extensions remains to be elucidated for both proteins, especially taking into account their high density of charged amino acids. Similar to the definition of specific motifs above, the precise determinants of RNA- vs. DNA-recognition and motif preferences will be best understood on the residue- or atom-resolved level, e.g. using NMR.

In this study, we provide the near-complete backbone assignment of the ARID domain of *Arid5a* including its N- and C-terminal elongations. With respect to their central role in specific nucleic acid-recognition, we also provide assignments of 6 out of 7 sidechain amides. We clearly define the core domain boundary and show that extensions do not interfere with the fold and remain flexible in the absence of nucleic acids. The herein provided chemical shifts serve a desirable starting point for follow-up structural and functional investigations on *Arid5a* alone and in complex with DNA and RNA, which will ultimately reveal its specificity on the transcriptional and post-transcriptional level.

## Methods and experiments

### Construct design

**Fig. 1:**
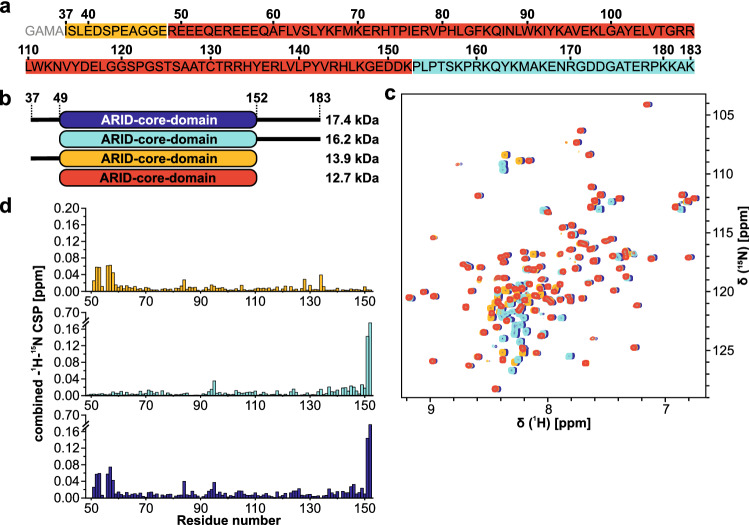
Constructs of *Arid5a* used in this study. **a** Sequence of *Arid5a* 37-183 numbered according to Uniprot entry Q03989. Non-native amino acids “GAMA” at the N-terminus are colored in gray. Red, orange and turquoise coloring of sequence corresponds to constructs in **b** Scheme of the four *Arid5a* constructs used in this study with their ARID core domain and extending IDRs (black lines). Respective molecular weights are shown on the right. **c** Overlay of ^1^H, ^15^N-HSQC spectra of the four *Arid5a* constructs. The spectrum of 37–183 is shifted slightly to the right for better visibility. Spectra are colored according to **b**. **d** Shift distance plots for the ARID between residues 49-152 of the three extended constructs (color-coded as in **b**) versus the core-construct (49-152). CSPs have been calculated in CCPNMR Analysis 2.5.2 (Vranken et al. 2005) with default settings

This study uses the human *Arid5a* amino acid (aa) sequence based on the UniProt (Bateman et al. 2021) entry Q03989 as shown in Fig. 1a. Domain boundaries were designed to comprise either the *Arid5a* core ARID (residues 49-152 of the full-length protein sequence) guided by the high sequence similarity (72.3%) to the second member of the *Arid5* subfamily, *Arid5b* [PDB ID: 2OEH, (Cai et al. 2007)], or N- (37-152) and C- (49-183) terminal elongations to allow for potential additional helices flanking the core domain (Korn and Schlundt 2022). Furthermore, a construct containing both extensions (37-183) was used to investigate possible structural influences on the core domain or with each other (Fig. 1b). For bacterial expression, the DNA sequence coding for *Arid5a* residues 37–183 was obtained from Eurofins Genomics, optimized for *E. coli* codon usage, and sub-cloned into the pET24d-derived vector pET-Trx1a (Gunter Stier, EMBL/BZH Heidelberg)(Bogomolovas et al. 2009; Peti and Page 2007) using oligonucleotides 5′-CGATTACCATGGCAATTAGCTTGGAAGATTCGCC-3′ (37_fwd) and 5′-GTGGTGCTCGAGCTATTTCGCTTTCTTCGG-3′ (183_rev), including an *NcoI* or *XhoI* restriction site, respectively. pET-Trx1a contains an N-terminal His_6_-tag and a thioredoxin-tag (Trx) followed by a tobacco etch virus (TEV) protease cleavage site. The three shorter variants of ARID (i. e. the core and the two individually extended versions) were cloned accordingly, using the template of *Arid5a* 37–183 and the additional following oligonucleotides: 5′-CGATATCCATGGCACGCGAGGAAGAGCAGGAACGGG-3′ (49_fwd) and 5′-GTGGTGCTCGAGCTATTTGTCATCTTCGCC-3′ (152_rev).

This construct design resulted in all four proteins containing four artificial N-terminal residues after TEV-proteolytic cleavage, termed Gly33/Gly45, Ala34/Ala46, Met35/Met47, Ala36/Ala48 for the natural termini 37 and 49, respectively.

### Protein production and sample preparation

*Arid5a* constructs were expressed in *E. coli* strain BL21(DE3) in M9 minimal medium containing 1 g/L ^15^NH_4_Cl (Cambridge Isotope Laboratories) and 4 g/L α-D-glucose (for ^15^N-labeled protein) or 2 g/L ^13^C_6_-D-glucose (Eurisotop) (for ^13^C/-^15^N-labeled protein) supplemented with 50 µg/ml kanamycin. All proteins were expressed for 16–20 h at room temperature after induction at an OD_600_ of 0.6–0.8 with 1 mM isopropyl-β‐d‐thiogalactopyranoside. After expression cells were harvested by centrifugation for 10 min at 4 °C and 6000×*g*. Cell pellets were resuspended in 50 mM Tris pH 8, 300 mM NaCl, 10 mM imidazole, 2 mM β-mercaptoethanol and 100 µL protease inhibitor mix G (SERVA) (25 ml per 1 L of cell culture). Cells were disrupted by sonication and the lysate was cleared by centrifugation for 20 min at 20,133×*g* and 4 °C. The cleared lysate was passed over a Ni^2+^-NTA gravity flow column (Sigma-Aldrich) and the protein of interest eluted with an imidazole concentration between 150 and 300 mM. The His_6_-Trx-tag was cleaved while dialyzing over night at 4 °C into fresh buffer without imidazole with 0.5 mg TEV protease (produced in-house) added per 1 L of culture (Tants et al. 2022). The cleaved tag and TEV protease were separated from the protein of interest by a second Ni^2+^-NTA gravity flow column, and the *Arid5a* constructs were finally purified by size exclusion chromatography (SEC) on a HiLoad 16/600 SD75 column (GE Healthcare) in SEC buffer (20 mM Bis-Tris, 1 M NaCl, 2 mM TCEP, 0.02% NaN_3_, pH 6.5). Purity of *Arid5a* containing fractions was determined by SDS-PAGE, and fractions containing pure *Arid5a* were pooled and concentrated using Amicon® centrifugal concentrators (molecular weight cutoff: 10 kDa). Finally, SEC buffer without NaCl was added to adjust the NaCl concentration to 150 mM, and the protein solution was concentrated for subsequent NMR measurements.

### NMR experiments

All NMR experiments (Table 1) were performed at the Frankfurt BMRZ using Bruker Avance III/Avance Neo spectrometers of 600, 700, 800 and 950 MHz proton Larmor frequency, equipped with cryogenic probes and using Z-axis pulsed field gradients. Measurements were performed at 298 K. For data acquisition and processing we used Topspin versions 3 and 4. For the assignments of the backbone and Asn/Gln/Trp side chains we used the following triple-resonance experiments: HNCA, HNCO, HNCACB, HN(CA)CO and CBCA(CO)NH. Additionally, we assigned the resonances of Hα and Hβ atoms for the core domain (49-152) in HBHANH and HBHA(CO)NH spectra. The {^1^H}-^15^N heteronuclear (het)NOE experiment of *Arid5a* 37-183 was measured as an interleaved pseudo-3D TROSY-based spectrum according to (Lakomek et al. 2012) with a saturation delay of 5 s at a concentration of 353 µM. All NMR experimental details are given in Table 1.

**Table 1:** Summary of acquired NMR experiments, parameters and samples

	Pulse program	c (µM)	TD (points)			SW (ppm)			MHz	NS	Labeling
			t1	t2	t3	F1	F2	F3			
49–152											
^15^N-HSQC	fhsqcf3gpph	330	128	2048	–	28	16	–	600	8	^15^N
^15^N-HSQC	fhsqcf3gpph	70	128	2048	–	32	16	–	600	32	^15^N
HNCACB	hncacbgpwg3d	127	90	58	1536	70	30	16	600	64	^15^N/^13^C
HNCACB	hncacbgpwg3d	471	100	62	1536	62	30	16	600	56	^15^N/^13^C
HNCA	hncagpwg3d	127	48	64	2048	30	30	14	700	48	^15^N/^13^C
HNCO	hncogpwg3d	127	48	64	2048	30	30	14	700	16	^15^N/^13^C
HN(CA)CO	hncacogp3d	471	50	64	1536	20	28	16	950	144	^15^N/^13^C
CBCA(CO)NH	cbcaconhgpwg3d	225	88	64	1024	70	30	16	800	36	^15^N/^13^C
HBHANH	hbhanhgpwg3d	225	80	64	2048	16	29	16	600	60	^15^N/^13^C
HBHA(CO)NH	hbhaconhgp3d	225	80	62	2048	11	29	16	600	52	^15^N/^13^C
49–183											
^15^N-HSQC	fhsqcf3gpph	70	128	2048	–	32	16	–	600	32	^15^N
HNCACB	hncacbgpwg3d	700	92	60	1536	62	30	16	600	48	^15^N/^13^C
CBCA(CO)NH	cbcaconhgpwg3d	700	82	62	2048	62	30	16	600	32	^15^N/^13^C
37–152											
^15^N-HSQC	fhsqcf3gpph	70	128	2048	–	32	16	–	600	32	^15^N
HNCACB	hncacbgpwg3d	500	100	68	1536	59	30	18	700	40	^15^N/^13^C
37–183											
^15^N-HSQC	fhsqcf3gpph	70	128	2048	–	32	16	–	600	32	^15^N
^15^N-HSQC	fhsqcf3gpph	300	256	4096	–	30	12	–	600	152	^15^N
hetNOE	trnoeetf3gpsi3d	353	122	1706	–	30	12	–	950	116	^15^N

#### Extent of assignments and data deposition

**Fig. 2:**
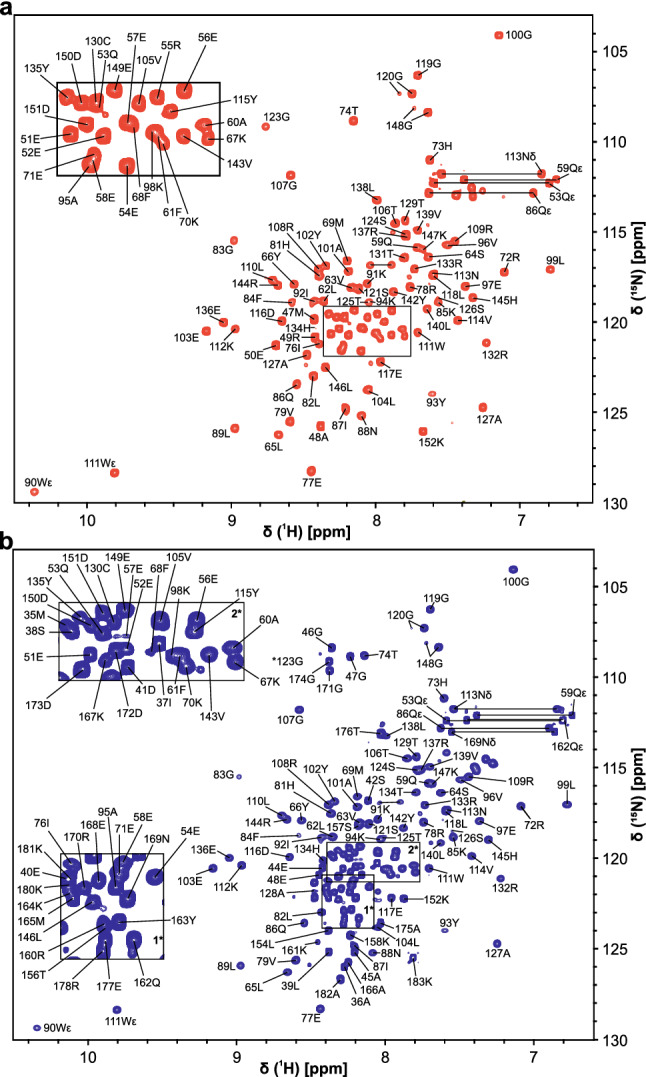
^1^H, ^15^N-HSQC-spectra of the ^15^N-labelled *Arid5a* 49-152 (**a**) and 37-183 (**b**) ARID domain constructs at 0.33 and 0.35 mM concentration, respectively, in 20 mM Bis-Tris pH 6.5, 150 mM NaCl, 2 mM TCEP, 0.02% NaN_3_, 5% (v/v) D_2_O collected at 298 K on a 600 MHz Bruker Avance III HD spectrometer equipped with a triple-resonance QCl cryogenic probe. Straight lines indicate side chain amide pairs. Tryptophan side chain amides are indicated with ε. Zoom-ins of spectral regions in boxes 1 and 2 are shown for clarity

The N- and C-terminal elongations to the ARID core domain had no significant influences on the fold of the core as shown in the overlay of the four ^1^H, ^15^N-HSQC spectra as shown in Fig. 1c and d. Similarly, the comparison of spectral overlay suggests no interaction of the extensions with each other. Thus, the assignments of ARID 37-152, 49-152 and 49-183 were performed separately using the CCPNMR Analysis 2.5 software (Vranken et al. 2005) and finally transferred to *Arid5a* 37-183 in order to obtain assignments of the fully extended *Arid5a* ARID domain. Figures of the spectra were created using the program Sparky (Lee et al. 2015). According to the ^1^H, ^15^N-HSQC spectra of *Arid5a* 49-152 and 37-183 shown in Fig. 2, both proteins represent a well-folded species judged by the broad spectral dispersion. However, regions highlighted for *Arid5a* 37-183 in insets 1 and 2 (Fig. 2b) show a considerable number of overlapping or less-resolved peaks clustering around 8 ppm (^1^H), and their resonances are assigned mainly to the N- and C-terminal parts of the protein sequence. The lack of dispersion hints at these regions being unstructured. The overall good quality of the spectra allowed for the backbone amide (^15^N-^1^H^N^) assignment of 99 and 97% for *Arid5a* 49-152 and 37-183, respectively (Table 2). Missing amide assignments locate to the glutamate/arginine (RE)-rich region (for *Arid5a* 37-183) at the N-terminal part (R49, E50, R55) and W90. Possibly, the missing amides of amino acids R49, E50 and R55 overlap with other glutamate or arginine peaks within the RE-rich region (see Fig. 1a) and are thus not assignable in the extended construct. Additionally, we assigned the following side chain residues: Q53ε, Q59ε, Q86ε, N88δ, W90ε, W111ε, Q162ε, N169δ. We found second, minor populations of G120 and G148 as unambiguously assignable. We suggest G120 senses cis and trans states of the adjacent P122. For *Arid5a* 37-183, we noted additional minor peaks, which, however, could not be unambiguously assigned. The chemical shifts of ^1^H, ^13^C and ^15^N backbone resonances for *Arid5a* 49-152 and 37-183 are available at the BioMagResBank (BMRB, https://www.bmrb.wisc.edu) under accession numbers 51811 and 51812, respectively.

**Table 2:** Completeness of assignments shown in this study. Fractions of NH assignments refer to non-proline residues, while assignments of the other nuclei refer to all residues

	Atom	CO	Cα	Cβ	Hα	Hβ	N-H^N^
% assigned	49–152	100	100	98	99	98	99
	37–183	–	98	97	–	–	97

**Fig. 3:**
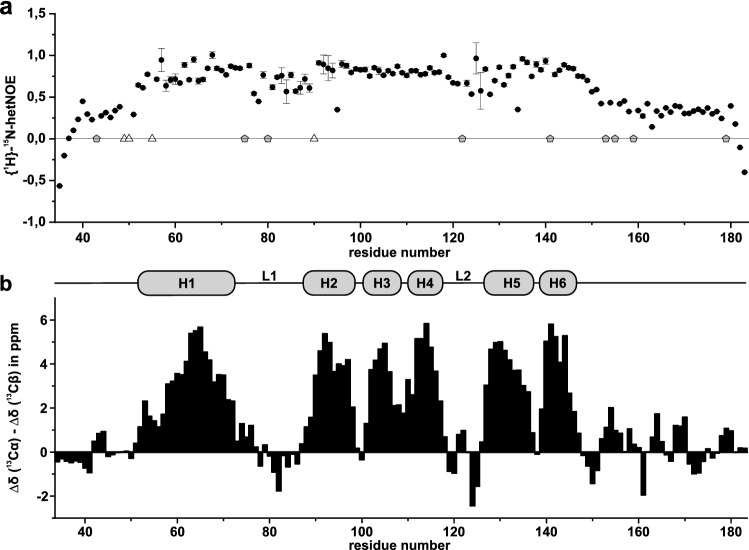
Display of {^1^H}-^15^ N heteronuclear NOE values (**a**) and combined Cα/Cβ carbon secondary chemical shift (SCS) values of *Arid5a* 37-183 plotted against the primary protein sequence as described by (Metzler et al. 1993) (**b**). **a** hetNOE values are shown with errors as derived from the program CCPNMR Analysis 2.5.2 (Vranken et al. 2005). Pentagons indicate prolines, and triangles indicate unassigned amino acid amides. **b** SCS for *Arid5a* ARID are shown with their interpreted secondary structure (shown above). α-helices are marked H1-H6 and indicated by gray bars above the SCS values and primary sequence. Loops L1 and L2 are shown for orientation

To further characterize the ARID domain’s N- and C-terminal extensions towards their structural content, we recorded a {^1^H}-^15^N-hetNOE experiment for ARID 37-183 to probe the dynamics of the protein on a fast time scale (Fig. 3a). This experiment reveals a large degree of rigidity between amino acids 54 and 149 (hetNOE values above 0.7). These findings are in line with the domain boundaries in the structure of *Arid5b* ARID (Cai et al. 2007), and also consistent with the high sequence similarity between *Arid5a* and *Arid5b*. Additionally, the hetNOE experiment revealed the presence of two flexible loops, L1 (aa 73-87) and L2 (aa 118-125), which show slightly lower ratios than the amides in adjacent helices. Furthermore, the N- and C-terminal parts to the ARID core domain show hetNOE values between 0.25 and 0.5, which indicates these regions are less rigid than the core domain, but not completely devoid of order.

The secondary chemical shift (SCS) plot in Fig. 3b was created by merging the values for Cα and Cβ values from HNCACB/CBCA(CO)NH spectra measured for *Arid5a* 49-152 (using aa 58-144), 37-152 (aa 34-57) and 49-183 (aa 145-183). From the SCS plot we interpreted the values towards the secondary structure of *Arid5a* ARID, where four consecutive residues with a positive value larger than 1 were defined as an α-helix, and three consecutive residues with a negative value below − 1 as a β-strand (Wishart and Sykes 1994). We thus confirmed the presence of six helices in the core domain, and the SCS plot also supports the extended L1 between helices 1 and 2. Helices 2, 3 and 4 are separated by only 2–3 amino acids each. H4 is followed by a second, shorter loop (L2). The C-terminal helices H5 and H6 are again separated by only 2 amino acids. In line with other ARID domains, the DNA-binding HTH motif is most likely provided by H4-L2-H5 (Cai et al. 2007; Iwahara and Clubb 1999; Kim et al. 2004).

In conclusion, we here provide the near complete backbone assignment of the ARID domain of *Arid5a* with its N- and C-terminal extensions. We unambiguously provide evidence, that the ARID domain is not extended by additional helices N- or C-terminally to the core domain but represents a “minimal” core domain type of an ARID. However, the highly charged extensions are likely to carry a decisive role in nucleic acid binding towards affinity and specificity. The herein obtained assignments provide the basis for a subsequent in-depth characterization of the domain towards its discriminative interactions with DNA and RNA on the atom-resolved level. Thus, our study is an important contribution towards understanding the structure-based target specificities of a DRBP member of the *Arid* protein family with essential roles in gene regulation and inflammatory and autoimmune diseases (Nyati et al. 2019).

## Data Availability

The chemical shifts of ^1^H, ^13^C and ^15^N backbone resonances for *Arid5a* 49-152 and 37-183 are available at the BioMagResBank (BMRB, https://www.bmrb.wisc.edu) under accession numbers 51811 and 51812, respectively. All NMR raw data used and analyzed during the current study are available from the corresponding author on reasonable request.
